# Poor Birth Outcomes in Malaria in Pregnancy: Recent Insights Into Mechanisms and Prevention Approaches

**DOI:** 10.3389/fimmu.2021.621382

**Published:** 2021-03-15

**Authors:** Caroline L. L. Chua, Wina Hasang, Stephen J. Rogerson, Andrew Teo

**Affiliations:** ^1^School of Biosciences, Taylor's University, Subang Jaya, Malaysia; ^2^Department of Medicine at Royal Melbourne Hospital, Peter Doherty Institute, University of Melbourne, Melbourne, VIC, Australia; ^3^Lee Kong Chian School of Medicine, Nanyang Technological University, Singapore, Singapore

**Keywords:** low birthweight, preterm birth, small for gestational age, malaria, pregnancy, VAR2CSA

## Abstract

Pregnant women in malaria-endemic regions are susceptible to malaria in pregnancy, which has adverse consequences on birth outcomes, including having small for gestational age and preterm babies. These babies are likely to have low birthweights, which predisposes to infant mortality and lifelong morbidities. During malaria in pregnancy, *Plasmodium falciparum-*infected erythrocytes express a unique variant surface antigen, VAR2CSA, that mediates sequestration in the placenta. This process may initiate a range of host responses that contribute to placental inflammation and dysregulated placental development, which affects placental vasculogenesis, angiogenesis and nutrient transport. Collectively, these result in the impairment of placental functions, affecting fetal development. In this review, we provide an overview of malaria in pregnancy and the different pathological pathways leading to malaria in pregnancy-associated low birthweight. We also discuss current prevention and management strategies for malaria in pregnancy, and some potential therapeutic interventions that may improve birth outcomes. Lastly, we outline some priorities for future research that could bring us one step closer to reducing this health burden.

## Introduction

Global efforts to combat malaria have resulted in declining malaria transmission in many regions over the last decade. Unfortunately, young children and pregnant women remain vulnerable, especially in areas with sustained transmission. Malaria, a disease caused by the *Plasmodium* spp. parasite, can result in severe morbidity and mortality. Worldwide, infection with *Plasmodium falciparum* contributes to the highest burden, and it will be the main focus of this review (1). Despite childhood exposure that leads to acquisition of immunity against malaria, first time pregnant mothers or primigravidas are again susceptible to the disease due to a combination of host and parasitic factors (2, 3). Malaria in pregnancy (MiP) results in placental infection, termed placental malaria (PM), which predisposes to placental injury and insufficiency. Placental insufficiency refers to poor placental function and is commonly observed in PM. It is also hypothesized to be a leading cause of low birthweight (LBW). A baby with LBW is defined as a live born who is <2,500 g regardless of gestational age (4). LBW deliveries can be due to preterm birth (live birth <37 gestational weeks) or small for gestational age (SGA) (birthweight <10th percentile for its gestational age). Of note, the precise mechanisms behind MiP-associated preterm birth and SGA remains unclear. *P. falciparum* infection can cause inflammation and potentially disrupt the fine immunological balance required to maintain pregnancy to term (5–7). On the other hand, SGA is often linked to placental insufficiency and there is also substantial evidence to suggest dysregulated placental development in mothers with MiP (8, 9). Interestingly, preterm birth does not commonly co-exist with SGA, further highlighting the complexity of MiP-associated birth outcomes (10).

LBW is an important indicator of infant mortality. MiP is estimated to cause approximately 900,000 LBW deliveries annually, with an estimated 100,000 MiP-related infant deaths (1, 11). Apart from high mortality risk, there is also increased morbidity in the surviving LBW infants, who are at elevated risk of poor cognitive and social development (12). LBW due to fetal growth restriction, the cause of SGA, is linked to increased incidence of adult diseases including type 2 diabetes and cardiovascular diseases (13). Hence, the prevention of MiP-associated LBW remains a priority in research. In this review, we discuss recent findings on the causes of LBW in *P. falciparum*-MiP and the different pathological pathways such as SGA and preterm birth in contributing to MiP-associated LBW. We also highlight existing gaps in our knowledge on the pathogenesis of LBW in MiP. Lastly, we review current interventions and provide suggestions for future work that allows us to better understand and manage MiP.

## Malaria in Pregnancy: An Overview of Pathogenesis and Immunity

**Figure 1:**
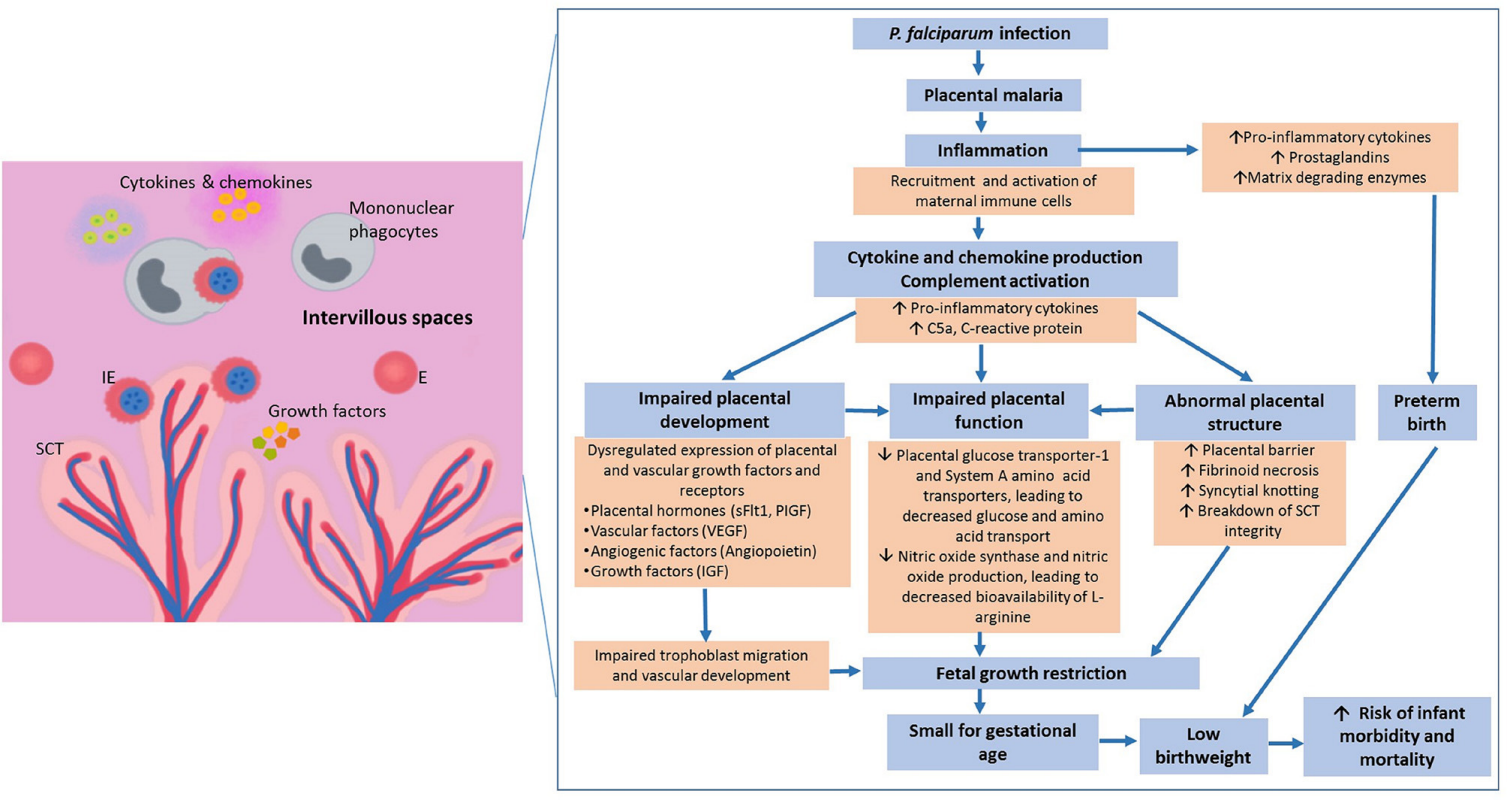
Pathways leading to malaria in pregnancy-associated low birthweight. Infected erythrocytes express unique variant surface antigens that mediate binding to placental receptors. Sequestration leads to recruitment and activation of different immune cells that can result in a cascade of downstream inflammatory events. These inflammatory events include secretion of cytokines and chemokines that have been associated with poor pregnancy outcomes. The increased pro-inflammatory environment has been associated with – (1) dysregulated expression of growth hormones, vascular factors and angiopoietin-1 and -2; (2) abnormal placental development; and (3) reduced placental nutrient transport, overall contributing to placental insufficiency. Together, these factors contribute to malaria in pregnancy associated low birthweight which is the biggest predictor of infant morbidity and mortality globally and annually., ↑, increase; ↓, decrease; E, Erythrocytes; IE, Infected erythrocytes; SCT, Syncytiotrophoblast; IVS, Intervillous space; sFlt1, soluble fms-like tyrosine kinase-1; PIGF, Phosphatidylinositol-glycan biosynthesis class F protein; VEGF, Vascular endothelial growth factor; IGF, Insulin-like growth factor.

Malaria in pregnancy threatens the well-being of the mother and her developing fetus, and an infected mother is likely to be an important reservoir of *Plasmodium* infection. One prominent feature of *P. falciparum*-infected erythrocytes (IEs) is their ability to adhere to endothelial and placental receptors. The latter allows them to sequester in the placenta, leading to placental infection and inflammation. Histological sections of *P. falciparum*-infected placentas reveal the presence of IEs, particularly at the surface of the syncytiotrophoblast, which is the main site of exchange for nutrient, waste and other metabolites between maternal and fetal circulations. Within the placental intervillous spaces, which are occupied by maternal blood, increased infiltrates of phagocytic cells with substantial amounts of ingested parasites can be observed; this is known as intervillositis (14, 15). The binding phenotype of IEs during pregnancy is mediated by their expression of variant surface antigens (VSA). Placental-binding parasites express VAR2CSA, a major VSA that is mainly expressed during pregnancy, and they bind specifically to the placental receptor known as chondroitin sulfate A (CSA) (16–19). Therefore, prior to pregnancy, antibodies against placental-binding IEs are uncommon, predisposing primigravidas to the adverse effects of PM (2, 20). In subsequent pregnancies, the protective anti-VAR2CSA antibodies can be naturally acquired in a gravidity-dependent manner and have been demonstrated to be effective against MiP and its consequences, thus preventing LBW (21). However, the specific antigenic targets and mechanisms of protection remain unclear. A recent systematic review showed that antibodies against different placental-binding antigens including VAR2CSA were associated with increased risk of PM and its consequences, suggesting that they may be markers of infection instead of correlates of protection (22). Nonetheless, it is likely that antibodies that are protective can inhibit binding of IEs to the placenta and/or facilitate clearance through opsonising phagocytosis, thus prevent placental inflammation and the subsequent adverse effects on fetal growth (Figure 1), and defining the characteristics of potentially protective antibodies remains a priority.

## Causes of MiP-Associated LBW

### The Link Between MiP, Dysregulated Placental Development and Small for Gestational Age Babies

Small for gestational age is commonly caused by fetal growth restriction. This condition significantly increases the risk of LBW, stillbirth, infant mortality and morbidity, and the risk of being diagnosed with chronic diseases in adulthood (13). Interestingly, SGA infants were two times more likely to experience *Plasmodium*-infection and clinical malaria by the age of 6 months, after adjusting for mosquito exposure (23). A recent study from an area of low malaria transmission reported several factors that were associated with increased odds of SGA during *P. falciparum*-related MiP, including symptomatic malaria and infection after 12–16 weeks' gestation (24), In addition, the risk of SGA was reported to increase by 1.13 times for every episode of infection during pregnancy (24). In contrast, in areas of high malaria transmission and particularly when pregnant women received anti-malarials in the form of intermittent preventive treatment in pregnancy (IPTp), there was no association between number of malaria episodes and SGA, suggesting that the timing of MiP may contribute to increased risk of SGA (25, 26). Indeed, placental pigment deposition (past-chronic infection) was associated with increased risk of SGA but not LBW or preterm birth, suggesting early chronic infection (usually outside of IPTp coverage) may drive SGA and not preterm labor (25, 27). Alterations in the downstream pathways of MiP such as growth factors and their regulators may further contribute to SGA. Recently, reduced plasma concentrations of placental growth factor in mid-pregnancy were associated with SGA and may indicate early placental insufficiency (9). MiP is known to be associated with reduced expression and bioavailability of placental growth factor, with the latter occurring through increased expression of their soluble inhibitor, soluble fms-like tyrosine kinase-1 (sFlt-1) (26, 28, 29). In addition, MiP leads to increased levels of asymmetric dimethylarginine, which is a competitive inhibitor of nitric oxide synthase (29). When nitric oxide synthase is inhibited, nitric oxide production is similarly affected, resulting in increased sFlt-1 levels and reduced levels of vascular endothelial growth factor (VEGF) family of proteins (30). This subsequently leads to dysregulated placental vasculogenesis and angiogenesis that may affect placental functions, contributing to SGA.

### The Link Between MiP, Inflammation and Preterm Babies

MiP is also associated with preterm birth, where these babies are usually born small and have increased risk of mortality due to complications such as brain hemorrhage, sepsis, acute respiratory illnesses and perinatal asphyxia (31). Surviving infants may develop life-long morbidities such as learning disabilities, which become obvious when the infant reaches school age (32). *Plasmodium* infection in both early and late pregnancies has been associated with preterm deliveries. A study from Malawi showed that earlier infection (before 24 weeks gestation) was associated with a higher risk of preterm delivery (26). In a Cameroon study, pregnant women who experienced MiP during the third trimester were four times more likely to have preterm deliveries (7). Similarly, in areas with low malaria transmission such as Southeast Asia, *P. falciparum* infection during late pregnancy was also associated with preterm delivery (24).

Studies on populations in malarious areas have identified several factors that significantly increase the risk of preterm birth, including PM, systemic inflammation (particularly CXCL9 and IL-1β), primigravidity, long-term iron supplementation and severe anemia (33–35). A recent systematic review found that maternal anemia in the first trimester, but not other trimesters, was associated with increased risk of preterm birth (36). Of note, the risk of MiP is highest during the first trimester, however, how severe maternal anemia contributes to preterm deliveries remains unclear. Interestingly, vertical parasite transmission, though uncommon, was also reported to contribute to preterm and LBW deliveries; this may partly be attributed to placental inflammation as higher complement activity was reported in cord blood infection (37, 38).

Several inflammatory mediators within the maternal peripheral circulation have also been associated with preterm delivery, but more investigations are required to understand whether they serve as biomarkers or play a pathological role. One of these markers is the soluble tumor necrosis factor receptor 2, which was found at increased levels in HIV positive women with MiP. Women who had elevated levels of this receptor throughout pregnancy also had preterm deliveries (6). Another study reported that in Papua New Guinean women who received one dose of IPTp with sulfadoxine-pyrimethamine (SP) and chloroquine, maternal serum levels of the acute phase protein α1-acid glycoprotein (AGP), an inflammatory marker, at delivery were positively correlated with several birth parameters including LBW, preterm and SGA deliveries (9). In the same study, women with two or more doses of IPTp with SP and azithromycin (SPAZ) were observed to have reduced levels of AGP at delivery, suggesting that SPAZ may reduce inflammation thus preventing deleterious pregnancy outcomes (9). The levels of AGP were previously shown to be increased in *P. falciparum*-infected children and adults with uncomplicated malaria illness (39, 40), while the role of AGP in MiP, especially in higher transmission areas, requires further investigation. Our understanding of the pathways leading to preterm birth in MiP is still rather limited, although placental inflammation is likely to be a main contributing factor. During the initiation of labor, a pro-inflammatory environment is generated through the activation of maternal immune cells and placental stromal cells, which subsequently produce high levels of pro-inflammatory cytokines, prostaglandins and matrix-degrading enzymes. These are associated with the triggering of uterine contraction, cervical ripening and membrane rupture, signaling the beginning of the birthing process. In MiP, particularly PM with intervillositis, the massive leukocyte infiltrates may trigger an inflammatory environment similar to the one observed in labor, leading to the premature initiation of parturition. Various cytokines that are implicated in the induction of preterm birth such as IL-1β, IL-6, macrophage migration inhibitory factor and CXCL1 have also been found at elevated levels during MiP (33, 41). Recently, in a murine model of MiP, dysregulated expression of placental ATP-binding cassette transporters was associated with preterm labor (42). Further studies are required to determine if a similar mechanism operates in the human disease.

## Potential Pathways Leading to MiP-Associated LBW

### Effects of MiP on Placental Development

The placenta is an important organ that acts as an interface between the mother and her fetus. Successful placental development requires a fine immunological balance to be achieved between maternal, placental and fetal immunity. PM can alter nitric oxide bioavailability, and it may lead to host inflammatory responses. Either of these processes may result in poor placental development, leading to placental insufficiency when the placenta is unable to fully support the growth of the developing baby (26, 29).

The first trimester is associated with the highest risk of *Plasmodium*-infection especially in primigravid women (43, 44). Critically, the timing of infection appears to determine the risks and severity of adverse birth outcomes. For MiP, early exposure (<15 gestational weeks) was associated with reduced volume of placental transport villi and smaller surface areas for diffusion across the villi (45). Other abnormalities such as impaired development of placental vascular space, excessive syncytial knotting (aggregation of syncytiotrophoblast nuclei), increased fibrinoid necrosis areas and thickening of syncytiotrophoblast basal membrane have also been observed (46–48). The timing of infection that resulted in such placental pathologies, however, is unknown. Ultrastructural studies showed the breakdown of syncytiotrophoblast integrity in regions associated with IEs (49). These abnormal changes to the placenta were associated with decreased birth weight (45, 46, 49).

MiP and the subsequent immune activation are associated with placental pathologies that arise due to disruptions in placental tissue (trophoblast) and vascular development. An *in vitro* model demonstrated impaired trophoblast invasion and migration after incubation with sera from *P. falciparum*-infected women (50). In the same study, elevated levels of soluble factors that inhibit trophoblast invasion such as human chorionic gonadotropin and IL-10 were found in these sera. There was a corresponding decrease in the invasion stimulatory factors such as insulin-like growth factors and IL-8 (50). There are two main events in vascular development; vasculogenesis (formation of new blood vessels) and angiogenesis (development of blood vessels from existing vasculature). In a recent review, three main pathways that are associated with impaired vascular development and adverse birth outcomes in MiP were identified, including reduced bioavailability of L-arginine and nitric oxide, excessive complement activation and dysregulated hemoglobin-scavenging system due to increased intravascular haemolysis (51). In a Brazilian cohort, MiP altered the angiogenic profile of the placenta and was associated with increased placental barrier thickness, which is likely to affect nutrient and gas transfer (47). Often, *Plasmodium*-infected placentas become the foci of inflammation where maternal immune cells are recruited and retained. This concomitant inflammation generates various soluble inflammatory mediators including tumor necrosis factor-α, interferon-γ, complement C5a, and C-reactive protein (33, 37). In addition, higher levels of chemoattractant proteins such as macrophage inflammatory protein-1 (alpha and beta) and monocyte chemoattractant protein-1 were observed (41, 52). These inflammatory mediators and chemoattractants are known to regulate the levels of key placental growth factors and receptors, including insulin-like growth factors, angiopoietin (1 and 2), soluble endoglin, sFlt-1 and VEGF (8, 37), which in turn disturb the delicate balance required for normal placental development. All of these analytes have been associated with reduced birth weight and LBW deliveries (33, 53). Taken together, placental trophoblast development, vascular development pathways and inflammatory responses appear to be interdependent and may synergistically contribute to poor birth outcomes in MiP. However, it remains to be determined if inflammatory inhibitors and/or promoters of placental growth factors may alleviate the impact of MiP on placental development and birth outcome.

### Intervillositis and Placental Insufficiency

Untreated *Plasmodium* infection during pregnancy increases the risk of intervillositis (inflammation in placental intervillous spaces). These spaces are occupied by maternal blood that surrounds the chorionic villi, serving as the materno-fetal exchange surface. Importantly, intervillositis, but not PM without intervillositis per se, was more commonly linked to adverse birth outcomes. This is particularly true in areas of high malaria transmission such as Tanzania and Malawi, where intervillositis was associated with abnormalities in the placenta and significantly higher rates of LBW (up to 50%) (15, 54, 55). PM-associated intervillositis is characterized by the accumulation of mononuclear cells, predominantly monocytes/macrophages. The inflammatory foci serve as a physical barrier that may compromise placental transport function and cause hypoxia. These immune cells can also be activated to produce inflammatory molecules that affect various downstream signaling pathways in the placenta, leading to impairment of fetal growth.

Investigations on *Plasmodium*-infected placentas revealed that compromised nutrient transport is one of the molecular mechanisms contributing to growth restriction and reduced birth weight. In women with PM-associated intervillositis, reduced expression of glucose transporter isoform 1 (GLUT-1) on the syncytiotrophoblast basal membrane was reported (56). Transplacental glucose transport is primarily facilitated by GLUT-1 and its expression was positively correlated with birthweight. In addition, lower expression and activity of system A group of amino acid transporters were also found in PM-associated intervillositis. Maternal to cord blood ratios of several amino acids were lower in cases with intervillositis compared to uninfected controls and importantly, cord concentrations of amino acids were positively correlated with fetal/placental weight ratios (57). In a follow-up study, lower placental mammalian target of rapamycin (mTOR) activity was observed in infected placentas with intervillositis, which in turn is associated with reduced placental amino acid uptake and lower birthweight (58). Recently, increased placental autophagy was proposed as a possible mediator of placental insufficiency in PM and similarly, this has been reported in other non-infectious pregnancy complications such as preeclampsia and intrauterine growth restriction (59–61). The dysregulation of this process may negatively affect the uptake of amino acids by the placenta and future studies should further explore this association.

## Prevention of MiP-Associated LBW: A Prospective on Current and Future Strategies

### Insecticide-Treated Nets, Indoor Residual Spraying, and Intermittent Preventive Treatment

**Figure 2:**
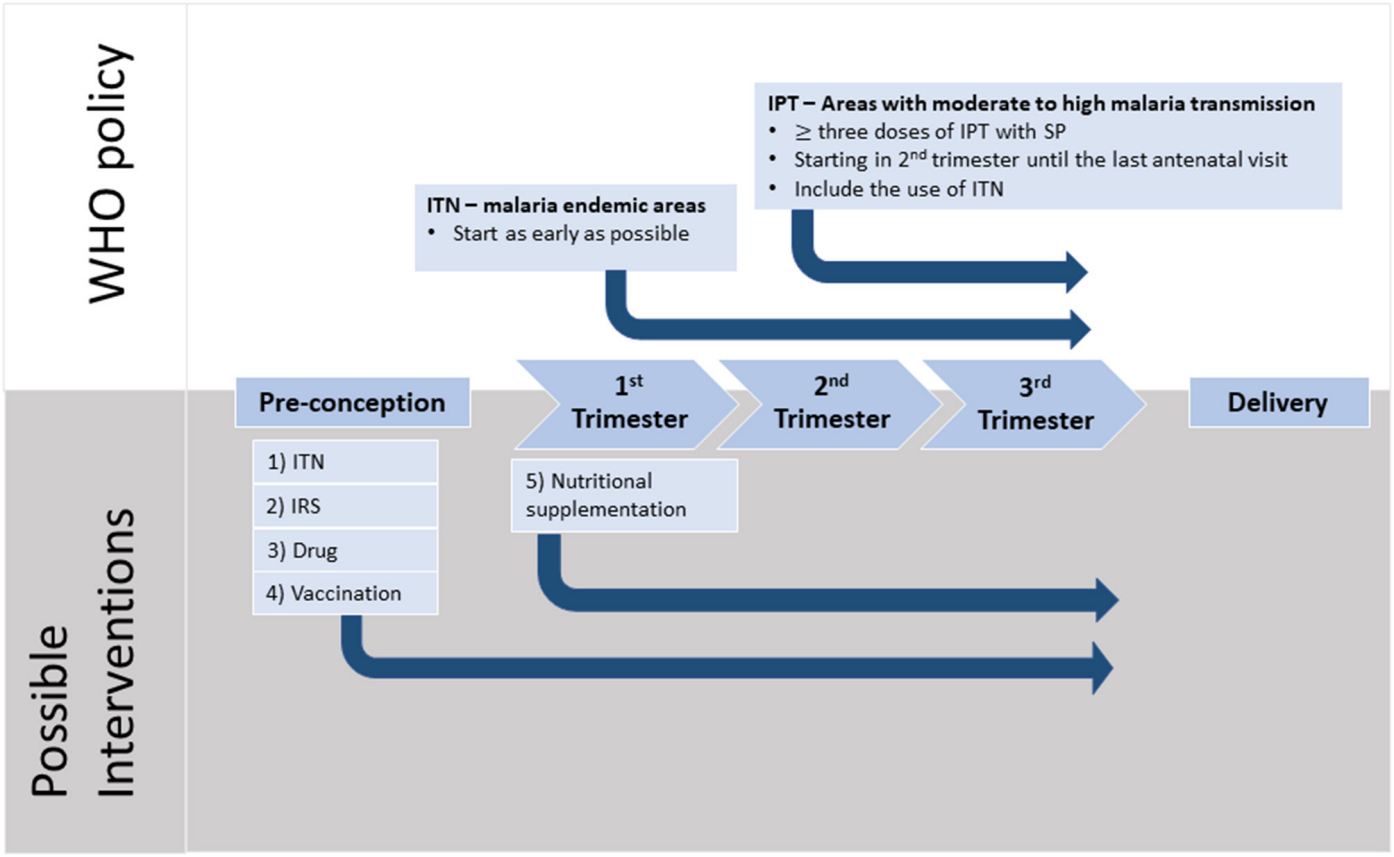
Timeline of pregnancy events with WHO recommendations and possible interventions. Malaria in pregnancy (MiP) is preventable and the current WHO recommendations for Africa include three or more doses of intermittent preventive treatment during pregnancy (IPT) with sulfadoxine-pyrimethamine (SP) taken one month apart starting from fetus quickening. In addition, sleeping under insecticide treated nets (ITNs) has been shown to reduce parasite burden during pregnancy. Strong evidence indicates that MiP starts during the first trimester with irreversible damage and it is during this period of time that pregnant mothers are at the highest risk of parasitemia. Furthermore, recent evidence suggests that women often harbor parasites before conception, and these parasites may switch to a placental-binding phenotype, that is likely to contribute to placental inflammation, especially in women pregnant for the first time. We propose several potential interventions which may help to alleviate the harmful impact of MiP. (1) ITNs should be used from early pregnancy, or before conception, to reduce the risk of acquiring new infections. (2) Indoor residual spraying should be evaluated for its effectiveness against MiP. (3) Pre-pregnancy intermittent screening for malaria, with treatment by safe and effective drugs, should be considered. (4) Pregnant women need access to an effective vaccine that can induce protection against MiP. Currently, there are two vaccines that are in clinical trials. (5) The nutritional status of the mother should be optimized during pregnancy with appropriate micro- or macronutrient supplements to ensure optimal placental development and thus better birth outcomes.

The World Health Organization recommends the prevention and management of MiP via the use insecticide-treated bed nets (ITN), IPTp and effective case management of malaria disease. Additionally, there are encouraging data to suggest that indoor residual spraying insecticide (IRS) may also be effective in reducing MiP and improving birth outcomes (62, 63). ITN and IRS, whether deployed individually or in combination, aim to reduce vector exposure and limit transmission, hence are likely to be beneficial (Figure 2). The use of ITN is a simple yet effective strategy to prevent *Plasmodium* infection and its consequences. There is substantial evidence from earlier studies suggesting that this strategy resulted in reduced prevalence of maternal parasitemia, improved birth weights and a 25% reduction in the risk of LBW deliveries (64–66). On the other hand, IRS, by decreasing vector contact, was associated with reduced risk of preterm birth, LBW delivery and fetal/neonatal deaths in both HIV-negative and HIV-infected women (62, 63).

Currently, all pregnant women residing in areas with high malaria transmission are recommended to receive three or more treatment courses of IPTp with SP, where each course should be administered at least one month apart, starting in the second trimester (67). This replaces the original recommendation of two doses of SP, following substantial data that demonstrated reduced risk of preterm birth and LBW as well as improved birth weight when three or more courses were administered (68–70). In mathematical modeling studies, it was estimated that three or more doses of IPTp-SP uptake would lead to a 30% relative reduction in LBW occurrence (70–73).

Despite these preventive strategies, the prevalence of PM and LBW remain a huge threat to pregnancies in malarious regions due to several reasons. Given that IPTp is only administered from the second trimester onwards, pregnant women are not protected during the first trimester and the later weeks of third trimester. This inadequate IPTp coverage throughout pregnancy, poor population coverage, development of drug and insecticide resistance, and the use of suboptimal drugs which results in the failure to clear submicroscopic infection, are likely to contribute to increased risk of preterm birth, SGA and LBW (26, 74, 75). Following reports on the increased prevalence of SP-resistant parasites, there are concerns regarding the future effectiveness of this prophylactic regimen in protecting against consequences of MiP (72, 76, 77). For this reason, other antimalarial drugs such as amodiaquine, mefloquine, chloroquine–azithromycin and dihydroartemisinin-piperaquine have been tested as potential alternatives to IPTp-SP (78, 79). Dihydroartemisinin-piperaquine appears to be a promising choice, as trial results showed that malaria burden including maternal parasitemia and PM was significantly lower in women who received this drug compared to those who received SP (80, 81). However, dihydroartemisinin-piperaquine did not lead to improved birth outcomes when compared with SP (81, 82). A recent systematic review showed that SP resistance decreased the effectiveness of IPTp especially in areas with high SP resistance, but this regimen is still associated with decreased cases of LBW (82).

The exact mechanisms by which SP leads to improved birth outcomes in the treated individuals remain elusive. In addition to malaria prevention, SP also serves as a broad-spectrum antibiotic that can resolve or prevent other infections of bacterial origin (sexually transmitted, reproductive tract and urinary tract infections) that have also been associated with adverse birth outcomes (69, 82). In addition, it was postulated that sulfadoxine can modulate bacterial microflora composition within the gut and vagina, which subsequently promotes weight gain (83) There is an ongoing study investigating the effect of SP on microbiota in the gut and/or vagina, in which the researchers proposed that higher birth weights following SP administration in pregnancy can be attributed to the modulation of these bacterial populations (84). To corroborate, antibiotic exposure during pregnancy led to increased gestational weight gain, which is an important predictor of birthweights (85). The overall reduction in malarial and other non-malarial infections during pregnancy is likely to minimize chronic maternal inflammation, thus improving birth outcomes (9, 82).

Given the limitations associated with current drugs, there is an urgent need to evaluate the safety and efficacy of new antimalarials or a combination of preventive strategies that are suitable for wider coverage and at earlier time points in pregnancy to protect against MiP in the first trimester.

### Nutritional Interventions a Viable but Under-Interventions: Approach

Another major contributor to LBW is maternal undernutrition, a condition that is also highly prevalent in many areas with high malaria transmission. There is a growing number of studies suggesting that malaria and maternal undernutrition may worsen pregnancy outcomes (86, 87). Malnourishment during pregnancy, indicated by low body mass index and smaller mid-upper arm circumference, was associated with lower levels of antibodies to placental-binding IE and increased parasite load in the placenta (88). The impaired development of protective antibodies may reduce the host's ability to clear infection, thus prolonged infection is likely to result in chronic inflammation in the placenta. In addition, undernutrition during pregnancy may also affect placental size and its functional capacity, given that maternal weight and gestational weight gain are both mediators of placental weight. All three factors were positively correlated to birthweight (89). Furthermore, both MiP and maternal undernourishment have been reported to impair trophoblast invasion and migration, as well as increase uterine artery resistance (50, 90, 91). In recent studies, MiP and undernourishment during pregnancy were observed to affect the levels of L-arginine-nitric oxide biogenesis; this pathway is essential for placental vascular development (29, 92).

In a comprehensive study that analyzed 23 systematic reviews on nutritional interventions during pregnancy, a few factors including provision of vitamin A, low-dose calcium, zinc and multiple micronutrients were shown to be associated with reduced risk of LBW (93). Similarly, several earlier macronutrient supplementation studies in malarious regions such as Sub-Saharan Africa provided evidence of improved birthweight, increased gestational length and reduced odds of LBW (94). Using a MiP animal model, dietary L-arginine supplementation resulted in improved fetal weights, reduced levels of pro-inflammatory mediators and increased levels of angiogenic factors, the last of which were associated with improved placental vascular development and remodeling (29). Nutrient intervention appears to be a promising strategy to prevent LBW, hence more studies should be carried out to evaluate the effectiveness of different types of nutrient supplementation in reducing MiP-associated LBW. Distinguishing between the potential beneficial impacts of malaria prevention with IPTp and of nutrient supplementation will require careful study designs.

### Vaccines: the Pivotal Prevention Strategy

Efficacious malaria vaccines could potentially be one of the most effective strategies to prevent LBW in MiP. Previous studies have described the requirements of an effective vaccine for MiP, which include the ability to induce protective antibodies against placental-binding IEs to prevent their binding to CSA in the placenta *via* the parasites' VAR2CSA protein. These binding-inhibition antibodies have been associated with protection against preterm birth, increased gestational age, the delivery of heavier infants and importantly, reduced risk of LBW delivery (21). Furthermore, these binding-inhibition antibodies against whole parasites were demonstrated to be strain-independent, raising the possibility that targeting an immunogenically-conserved subunit or domain of VAR2CSA, which is a large (~350 kDa) multidomain protein, could be effective (95). Given its large size, later studies then identified a minimal binding region on the VAR2CSA protein that binds with high affinity to CSA. Antibodies in the sera collected from malaria-exposed pregnant women were able to bind to recombinant VAR2CSA and purified antibodies against this protein exhibited binding-inhibitory activity (96–99). Importantly, a study from Benin then demonstrated strong antibody responses against the VAR2CSA N-terminal region of DBL3X to be associated with protection against SGA and reduced prevalence of both LBW babies and placental infection at delivery, possibly by reducing parasite burden and thus lowering the risk of placental insufficiency (97). However, recent studies have identified distinct pathogenicity among the parasite variants and diversity in functional antibodies against different placental binding variants, suggesting that a polyvalent VAR2CSA-based vaccine may provide better protection (100, 101). To corroborate, two isoforms of VAR2CSA subunit protein that recently completed phase 1 clinical trial, ID1-ID2a (PAMVAC) and DBL1x-DBL2x (PRIMVAC), only demonstrated binding-inhibition activities against homologous parasite variants and the duration of immune responses is unknown (102, 103). Critically, the duration of immune responses and whether vaccination translates to clinical protection remains to be determined.

## Future Directions

Great advances have been made over the last two decades in understanding and reducing MiP, especially with effective anti-malarials and the use of ITNs during pregnancy. Despite this, our understanding of factors that contribute to LBW during MiP is still rather limited. In addition, with the increasing pressure of parasite resistance against existing drugs, alternatives such as a viable vaccine will be crucial to combat MiP. Hence, more research efforts should be channeled toward these areas of investigation. Herein, some areas of future research are proposed.

1. To better understand and manage the adverse birth consequences of MiP, there is a need to differentiate the relative contributions of SGA and preterm delivery to LBW. SGA and preterm delivery in MiP may be initiated by different molecular events, and hence may require different modes of interventions. In resource-limited malaria endemic areas where ultrasound is unavailable, pregnancy dating is based on recall of last menstrual period, abdominal palpation, or newborn assessments, all of which may be inaccurate. In addition, the reference curve for birth weights is based on those that have been established in the Western populations. Several studies have created population-specific charts detailing mean birth weights and percentiles at different gestational ages to have a better representation of their own populations (10, 104). In addition, a global healthcare project has developed standards and tools for assessing newborn weight, length and head circumference by gestational age and sex (105). These can lead to more accurate classifications of birth outcomes in MiP and studies on their effectiveness in differentiating SGA and preterm birth can be done.
2. Changes to the placenta in PM are only studied upon delivery, given the invasive nature of sampling placental blood or biopsies during pregnancy. However, this rarely captures the whole picture of placental events that occurred throughout pregnancy, limiting our interpretation of how placental changes during gestation can lead to LBW. Although the use of animal models may address this challenge, there are concerns regarding the relevance and transferability of the findings to human disease (106). One way to better understand the placental pathophysiology during MiP is to employ the use of Doppler ultrasound to investigate utero-and feto-placental blood flow and resistance. In earlier studies, MiP in the first half of gestation was associated with altered uteroplacental blood flow, suggesting the possibility of vascular deficiency (90, 107). This technique, together with measurement of various maternal analytes in the peripheral blood, may provide a clearer picture of the events in the placenta during MiP.
3. Our current understanding on inflammation in PM focuses on the roles of monocytes and macrophages as the main contributors of inflammatory mediators associated with LBW. However, there are recent studies suggesting that neutrophils, which are the most abundant leukocytes in the peripheral blood, may also play a role in orchestrating the inflammation (108, 109). Their role in MiP requires further clarification.
4. Our current understanding of protective antibodies in MiP is that these antibodies should be able to inhibit IEs binding to the placenta and promote opsonic phagocytosis (21). However, their importance and relative contribution to protection through other immune effector mechanisms such as antibody-dependent respiratory burst and antibody-dependent cellular cytotoxicity are less well-understood. This knowledge will be important in vaccine design and the evaluation of vaccine efficacy.
5. Early studies in The Gambia and Nigeria demonstrated that passive transfer of anti-malarial antibodies resolved malaria (110, 111). Currently, there are existing clinical trials that are investigating the protective efficacy of monoclonal antibodies as anti-infective agents (ClinicalTrials.gov Identifier: NCT04327310 and NCT04206332). Therefore, monoclonal antibodies, targeting either the domains of VAR2CSA or the circumsporozoite protein to prevent establishment of infection can be further developed as therapeutics for MiP.
6. In terms of improving vaccine design, there is a need to identify conserved subdomains of VAR2CSA that can induce protective antibodies, against the different parasite variants.
7. SP may be less efficacious in areas with high SP-resistant parasites. Moreover, SP is only given at the start of second trimester, but there is substantial evidence showing that infection during the first trimester can also greatly impact birth outcomes (24, 26). Hence new antimalarial drug combinations that can be administered in the first trimester need to be evaluated in future clinical trials.
8. SP, a broad-spectrum antibiotic, may be effective against sexually transmitted and reproductive tract infections (highly prevalent in malaria endemic regions), thus it may resolve infections other than malaria to improve adverse birth outcomes (69). Future studies to explore the use of effective antimalarials coupled with SP may be beneficial.
9. Malnutrition is an important contributor to LBW, and nutrient supplementations during pregnancy appear to be an attractive and feasible intervention to minimize the risk of LBW deliveries. However, the beneficial effect of nutrient supplementation may be masked if administered together with IPTp, thus undermining its impact on birth outcomes. Hence, future trials of nutrient supplementations require careful study designs such as having intermittent-screening and treatment with nutrient supplementations, or choosing a trial site in a lower transmission setting where IPTp is not a first line policy.

## Conclusion

As of 2019, 38 countries and territories were granted the “malaria-free” status by WHO, while transmission continues in other regions. Hence, pregnant women remain at risk of MiP, which leads to increased mortality and morbidity in both the mother and her infant. MiP-associated LBW is preventable, but there needs to be a concerted effort in the adoption and implementation of current prevention strategies in malarious areas. Continuous efforts in research are needed, to better understand the molecular mechanisms associated with poor birth outcomes, and ultimately to enable design of specific targeted approaches for improving birth outcomes. In the face of increased parasite resistance, the effectiveness of IPT with SP in preventing this health burden may be at threat in the near future. Therefore, there is an urgent need to design and evaluate other therapeutic options that can be used to protect against MiP and its deleterious consequences.

## Author Contributions

All authors listed have made a substantial, direct and intellectual contribution to the work, and approved it for publication.

## Conflict of Interest

The authors declare that the research was conducted in the absence of any commercial or financial relationships that could be construed as a potential conflict of interest. The handling editor declared a past co-authorship with one of the authors SR.
